# A Novel Appointment Protocol to Accelerate Orthodontic Treatment: A Case Report

**DOI:** 10.7759/cureus.84808

**Published:** 2025-05-25

**Authors:** Uday Kumar Alle

**Affiliations:** 1 Dentistry and Orthodontics, SEHA, Ghayathi Hospital, Abu Dhabi, ARE

**Keywords:** accelerated orthodontics, archwire sequencing, case report, frequency of orthodontic appointments, orthodontic adjustments, orthodontic appointment interval, orthodontic archwires, self-ligating brackets

## Abstract

The need for orthodontic treatment among adult patients has been steadily increasing. A considerable number of these patients request to expedite their treatment owing to personal circumstances, including travel, relocation to another country, university enrollments, and preparations for weddings or other celebrations. To achieve expedited outcomes, various techniques have been devised over the years to expedite tooth movement during orthodontic treatment, encompassing pharmacological, surgical, and physical methods. Efficient scheduling is acknowledged as a crucial factor in the timely completion of orthodontic treatment. A uniform approach is not appropriate for scheduling orthodontic appointments. The optimal time frame varies depending on the specific contextual factors.

We have implemented a novel orthodontic appointment protocol that entails scheduling orthodontic adjustment appointments every 10 days and employing metal passive self-ligating brackets combined with rapid archwire sequencing from lightest archwires to thick archwires in a gradual approach starting with lighter archwires, which efficiently facilitated expedited treatment.

## Introduction

The optimal interval between orthodontic appointments has long been a subject of discussion, which is frequently influenced by clinician preference and institutional guidelines [1]. Similarly, the sequencing of archwires during orthodontic treatment is influenced by both the clinical judgment of the practitioner and the variations in protocols recommended by different manufacturers and various bracket systems. The evidence suggests that a uniform approach is not compatible with the scheduling of orthodontic appointments. The appropriate intervals may vary from one to two weeks to eight to 10 weeks, contingent upon factors including patient age, type of archwire and force-delivery system, periodontal condition, extraction versus non-extraction, surgical and impacted cases, compliance versus non-compliance mechanics, decalcification and white spot lesions, root resorption, and scheduling considerations [2]. Shorter intervals between appointments appear to contribute to effectively managing the treatment and result in shorter treatment times. The objectives and duration of treatment can be modified to attain the desired results [3].

Adult patients frequently request expedited orthodontic treatment owing to obligations such as professional travel, relocation to a different country, university enrollments and hostel stays, or an upcoming significant life event like weddings. The multiple visits to the orthodontist and prolonged treatment time, combined with the aesthetic challenges of fixed braces, wires, and appliances, often cause patient dissatisfaction and reluctance to initiate treatment. The literature indicates a lack of consensus on the average duration of orthodontic treatment and frequency of appointments. A systematic review reported an average treatment time of 19.9 months with fixed appliances; however, this varied significantly across studies, ranging from 14 to 33 months [4]. When evaluated according to the American Board of Orthodontics (ABO) standards, the mean treatment duration for single-phase orthodontic treatment was 24.6 months [5]. The treatment goals and timelines can be modified to achieve acceptable results based on satisfactory occlusal stability. A crucial step in managing treatment duration is the accurate identification of treatment objectives. The orthodontist should ascertain patients' expectations and treatment objectives, and the duration might be modified to achieve particular outcomes. Such objectives can be accomplished with sufficient occlusal stability [1].

This case report presents a novel protocol for orthodontic adjustment appointments that expedited treatment durations. The procedure employs passive metal self-ligating brackets (SLBs) and 10-day appointment intervals in synchrony with rapid archwire sequencing with a gradual increase in size starting with the lightest archwires, which are aimed at enhancing the efficiency of tooth movement and augmenting overall treatment efficacy. The gradual alteration of archwires with rapid sequence facilitated the attainment of working archwires in a short duration, owing to minimal force variation, with the least discomfort to the patient. This protocol attempts to minimize the total duration of orthodontic treatment while maintaining excellent standards of care and ensuring patient comfort through the optimization of scheduling and procedures during adjustment appointments. This method differs from orthodontic treatment with SLBs, which typically involves extended intervals between adjustment visits.

## Case presentation

Diagnosis and etiology

**Figure 1:**
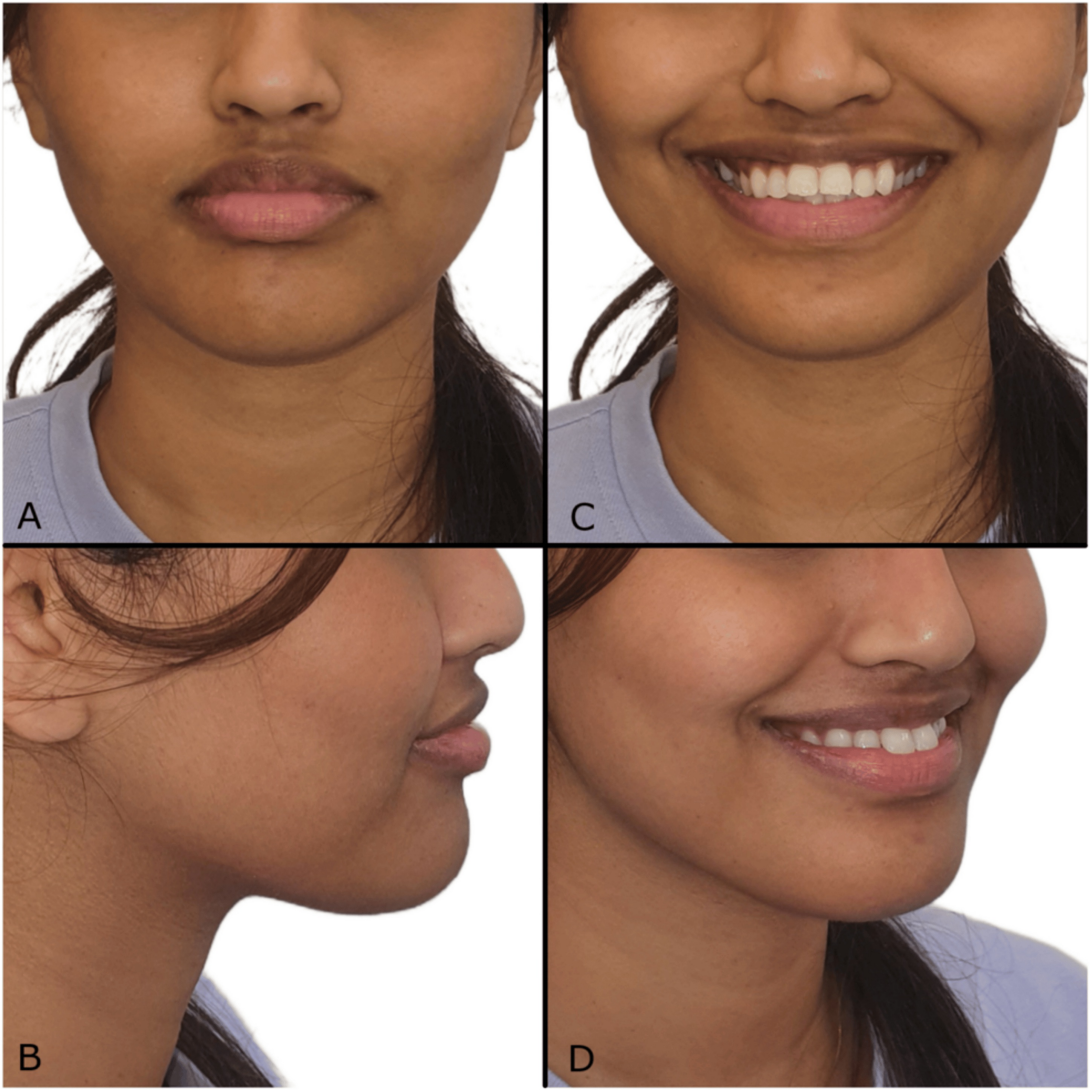
(A-D) Pre-treatment extraoral photographs

A 19-year-old medically fit female patient presented to the orthodontic clinic with the chief complaint of sticking-out top front teeth and expressed a desire to complete her treatment within three months owing to impending travel. The clinical examination revealed a significant non-consonant and reverse smile arc. There is a mild concave facial profile with a proportional lower third of the face, reduced nasolabial angle, and mild accentuated mentolabial sulcus. Extraoral photos revealed symmetrical facial proportions with average facial heights (Figure 1). The patient had protruding upper and lower lips, a normal ratio of upper and lower lips, decreased philtrum length, and an average buccal corridor area. Smile aesthetics on posed smile revealed reverse and non-consonant smile arc, 80% upper incisor display, and 20% lower incisor display. The upper dental midline was coincident with the facial midline, and the lower dental midline shifted to the left side by 1 mm with the upper midline. Intraoral soft tissue examination revealed satisfactory oral hygiene with normal gingiva and oral mucosa. Intraoral hard tissue examination revealed a complete set of erupted teeth, excluding all third molar teeth. An exaggerated curve of Spee was observed in the mandibular arch; the upper arch showed a convex curve.

**Figure 2:**
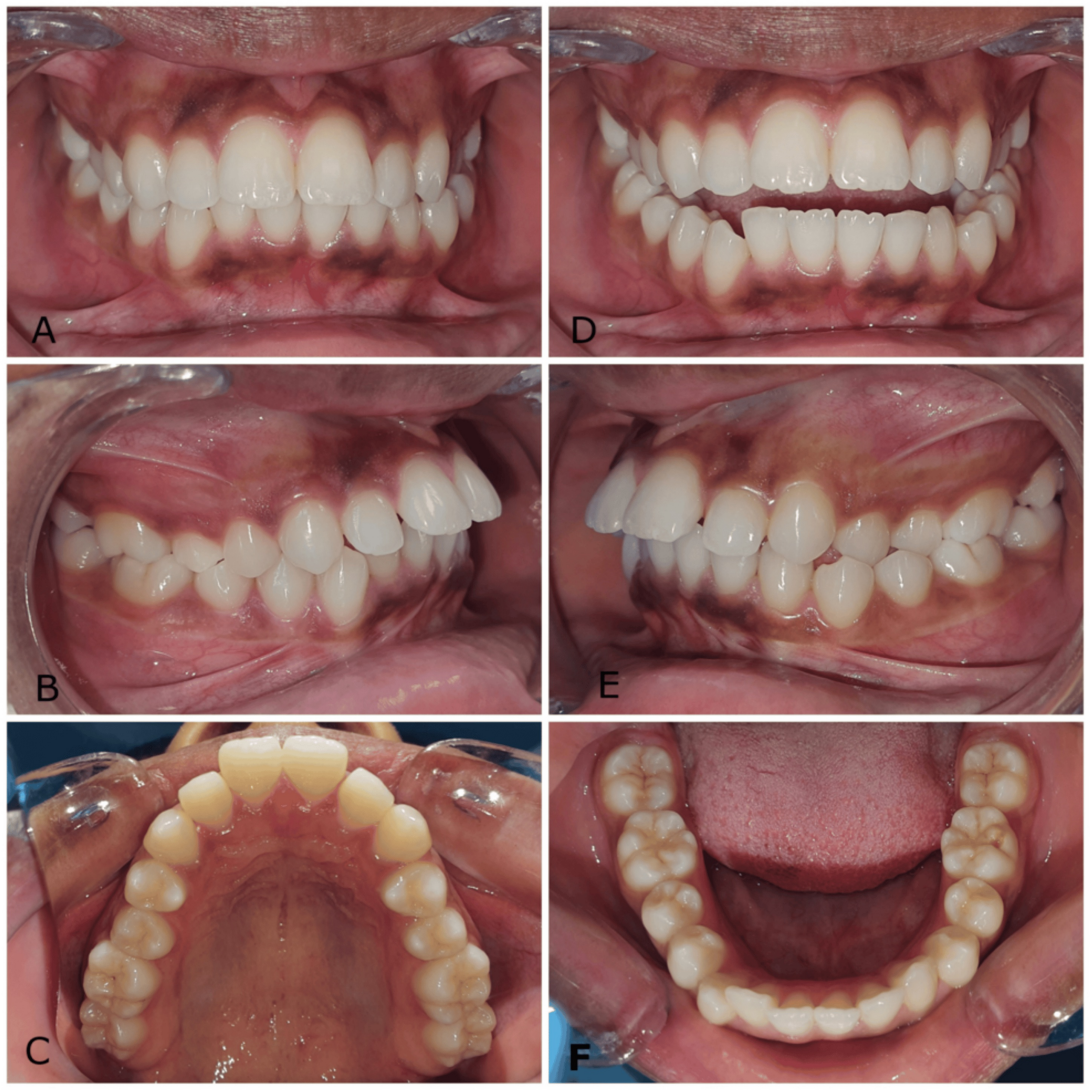
(A-F) Pre-treatment intraoral photographs

**Figure 3:**
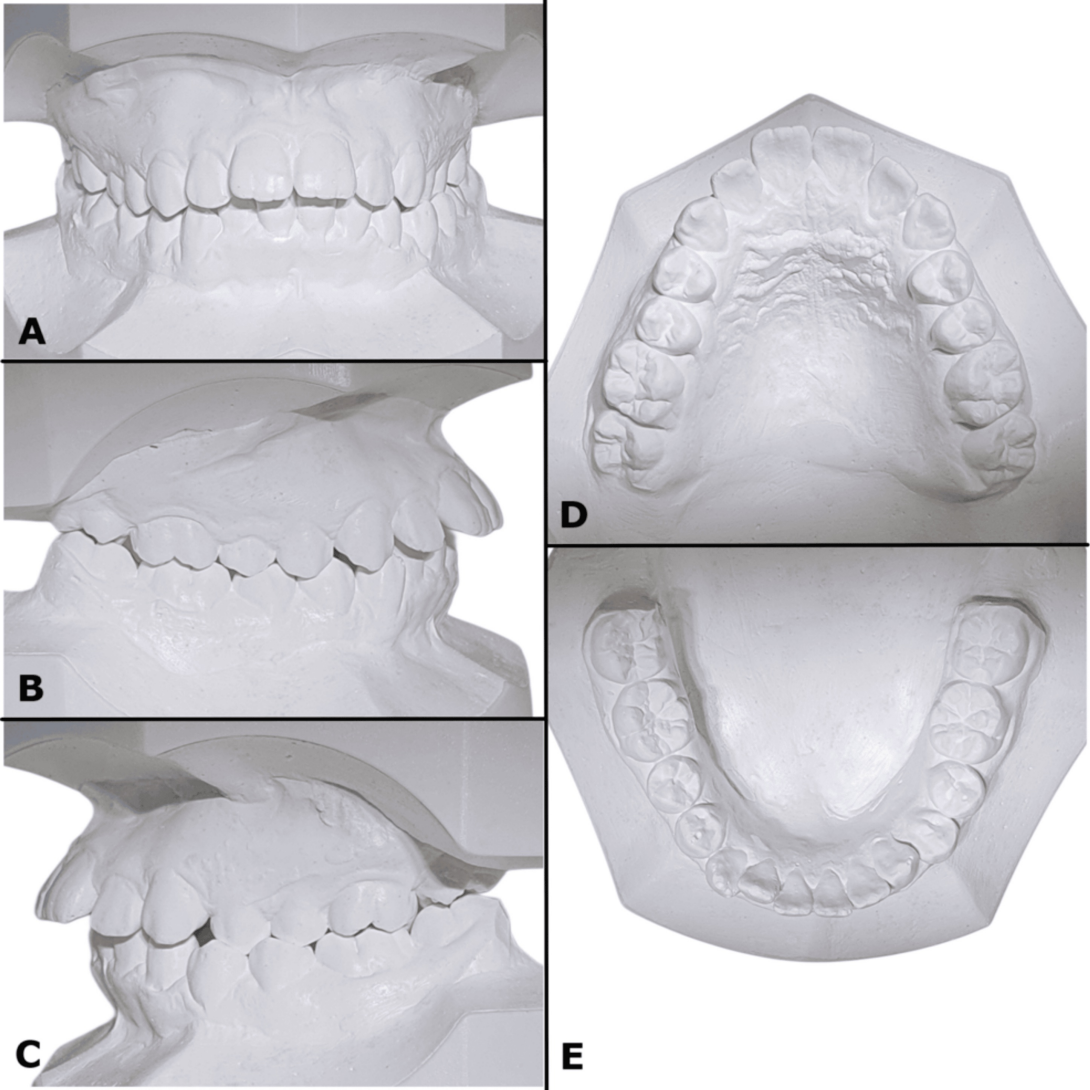
(A-E) Pre-treatment study models

Intraoral and dental study model examinations revealed a class I bilateral molar relationship and class II div 1 incisor relationship on a class I skeletal base with an average-sized maxilla and mandible. The right canines were in class I, and the left canines were in half-unit class II. The upper left first and second premolars were in lingual crossbite. The upper incisors were proclined with an overjet of 7.5 mm, and the lower anterior teeth exhibited mild crowding (Figures 2, 3).

**Figure 4:**
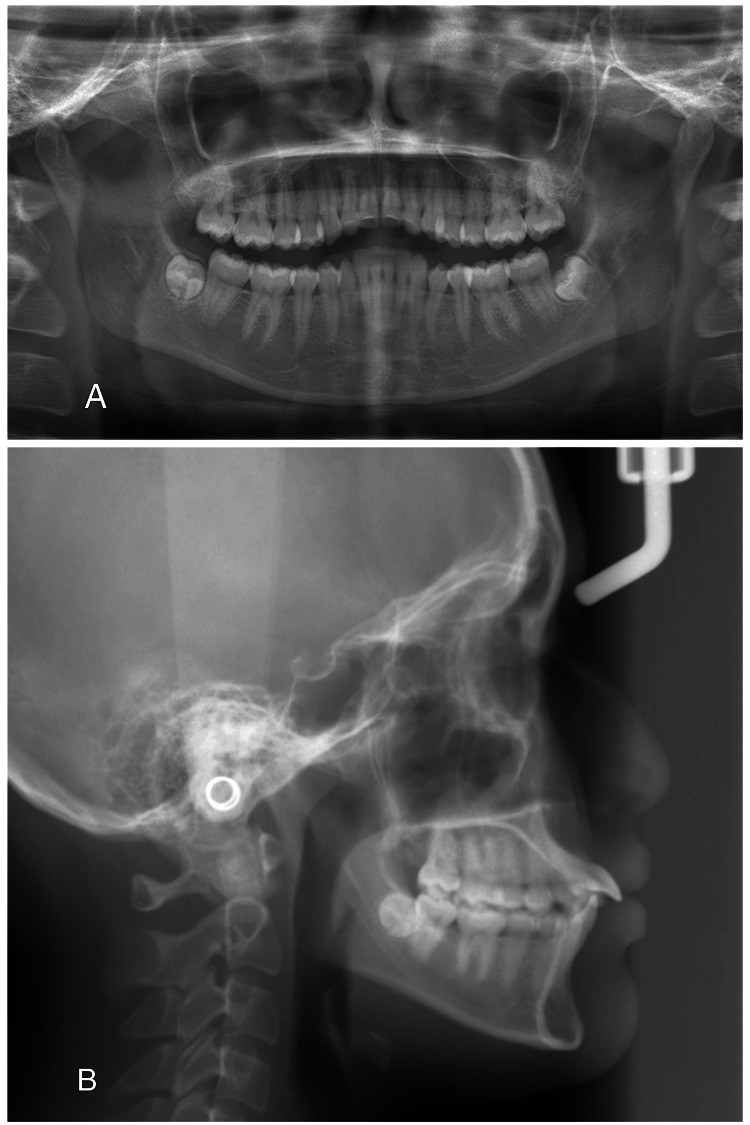
Pre-treatment radiographs: (A) panoramic radiograph and (B) lateral cephalogram

The panoramic radiograph revealed complete permanent dentition with developing lower third molar teeth and absent upper third molar buds. No abnormalities in bone and tooth morphology were noted, and there were no periodontal or periapical problems observed (Figure 4).

Cephalometric analysis revealed a skeletal class I relationship with the average maxilla (Sella-Nasion-A (SNA) 83°) and mandible (Sella-Nasion-B (SNB) 80°) and average growth pattern, normal mandibular plane angle, proclined maxillary incisors (U1-PP 136°), and average inclined mandibular incisors (incisor mandibular plane angle (IMPA) 96°).

Treatment objectives

The treatment objectives were to (1) establish and maintain optimal oral hygiene throughout the orthodontic treatment, (2) correct the upper left first and second premolars lingual crossbite, (3) correct crowding in the lower incisor region, (4) correct the curve of Spee through arch leveling and alignment, (5) correct the inclination of the upper incisor teeth, (6) correct the overjet, (7) maintain class I molar on the right and left sides and establish incisor and canine class I relationships, (8) achieve an aesthetic profile and consonant smile arc, and (9) preserve the achieved results through appropriate retention methods.

Treatment alternatives

In accordance with treatment objectives, the following alternatives were presented to the patient: (1) The patient was scheduled for regular monthly follow-up appointments to manage treatment using either conventional brackets or SLBs. The objective was to ensure continuity of care during her relocation, where the remainder of her orthodontic treatment would be completed. (2) Another option proposed involved clear aligners, allowing for the initiation of aligner therapy and the provision of subsequent progress trays. This approach aimed to support seamless treatment continuation and monitoring at her travel destination.

The patient was willing to wear orthodontic appliances for only three months, to be able to travel and pursue higher education abroad thereafter. On approval of the treatment plan and a complete explanation of the outcomes and other possibilities and side effects, the final plan was made. It included an improvement of maxillary incisor proclination and mandibular anterior teeth crowding, the correction of the curve of Spee, and an improvement of smile aesthetics within the stipulated duration utilizing metal passive SLBs. After obtaining all diagnostic records and discussing with the patient about recommended intervals, we implemented the new protocol for orthodontic adjustment, consisting of appointment intervals of 10 days, and informed the patient about possible results. To address financial constraints and the limited duration of the patient’s remaining stay, she requested that the treatment be completed within a three-month period. In response, we implemented a novel approach, scheduling orthodontic adjustment appointments at 10-day intervals. This condensed orthodontic appointment protocol was designed to optimize treatment outcomes within the restricted timeframe while maximizing therapeutic effectiveness.

Treatment progress

The treatment protocol involved a non-extraction orthodontic approach employing upper and lower pre-adjusted edgewise appliances (0.022" x 0.028" slot) with a metal passive SLB with a metal rigid sliding gate of MBT prescription (DTC Orthodontics, Glory II series, Mini Self-Ligating Brackets, Newark, DE, USA). Each orthodontic adjustment appointment with 10-day intervals involves a rapid archwire sequence with gradual archwire progression to the next possible archwire size.

**Figure 5:**
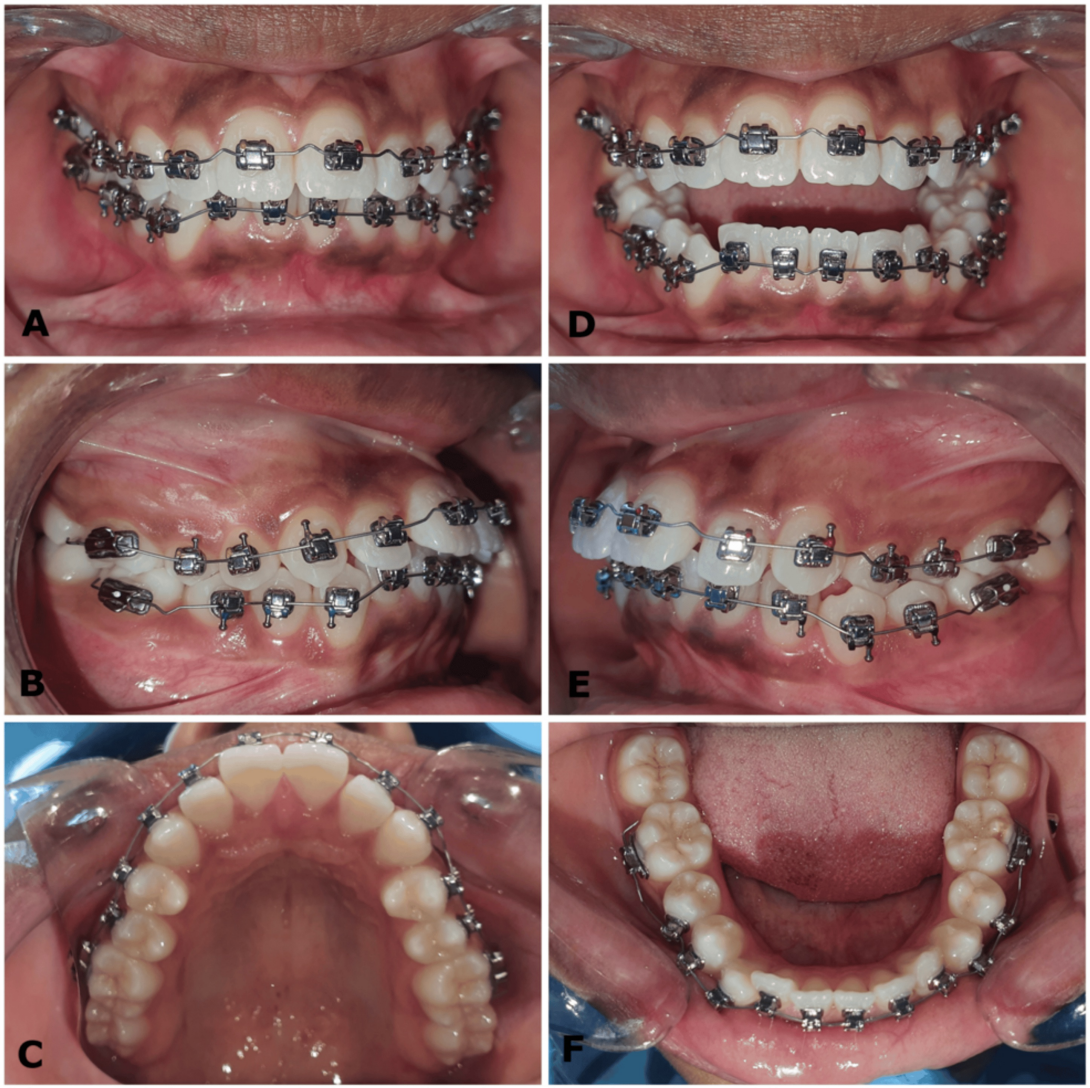
(A-F) Intraoral photographs show initial orthodontic bracket bonding and the placement of 0.012″ NiTi archwires with V bends in the upper and lower arches

By mid-September 2023, the case history, complete records (intraoral and extraoral photographs, diagnostic impressions), and panoramic and lateral cephalometric radiographs were completed. The patient was referred to a General Practitioner (GP) Dentist for oral prophylaxis. The upper and lower arches were bonded from the first molar to the first molar, excluding the second molars from the treatment plan due to the potential for bite opening and occlusal instability within the limited timeframe. Initial 0.012” thermal nickel-titanium (NiTi) archwires were inserted in upper and lower arches, with V bends incorporated utilizing V-bend pliers in archwires to enhance flexibility and mitigate archwire displacement (Figure 5).

**Figure 6:**
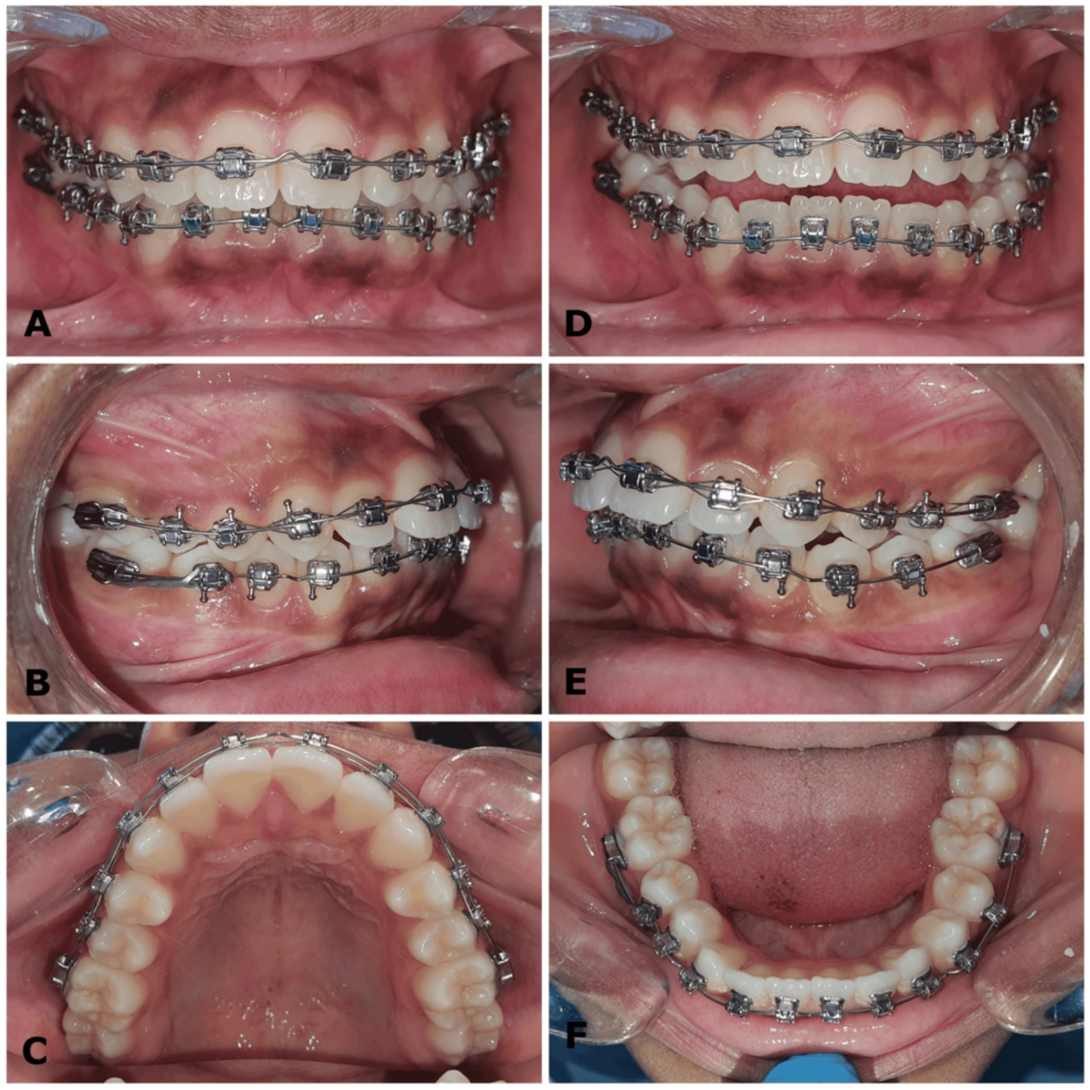
(A-F) Upper arch with 0.018 NiTi archwire, lower arch with 0.018 NiTi archwire with intrusion bends distal to canines

The upper and lower archwires transitioned from 0.012" thermal NiTi to 0.018" superelastic NiTi (0.012" thermal NiTi and 0.014”, 0.016", and 0.018" superelastic NiTi archwires). The lower arch 0.018" NiTi archwire had intrusion bends positioned distal to the canines using V-bend pliers (Figure 6).

**Figure 7:**
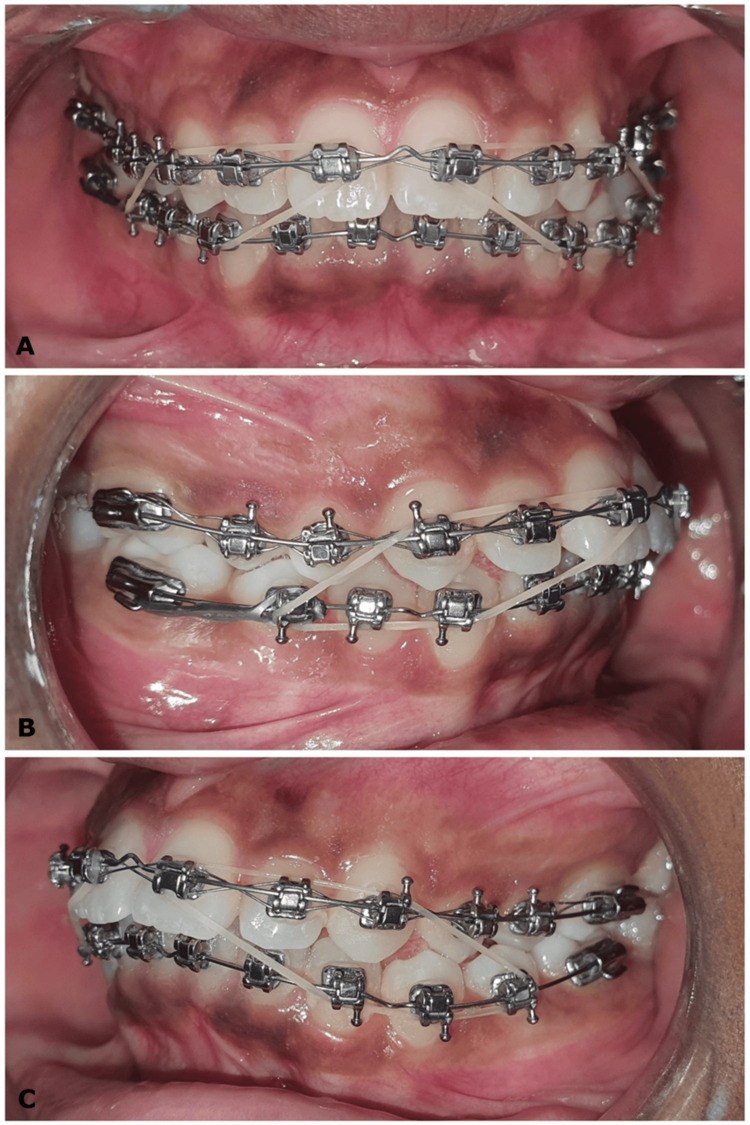
(A-C) Intermaxillary class II elastics of 1/4" size and 3.5 oz

In October 2023, upper incisor proclination correction was achieved utilizing short intermaxillary elastics 1/4" and 3.5 oz applied from lower premolars and canines to upper incisors and canines bilaterally (Figure 7).

**Figure 8:**
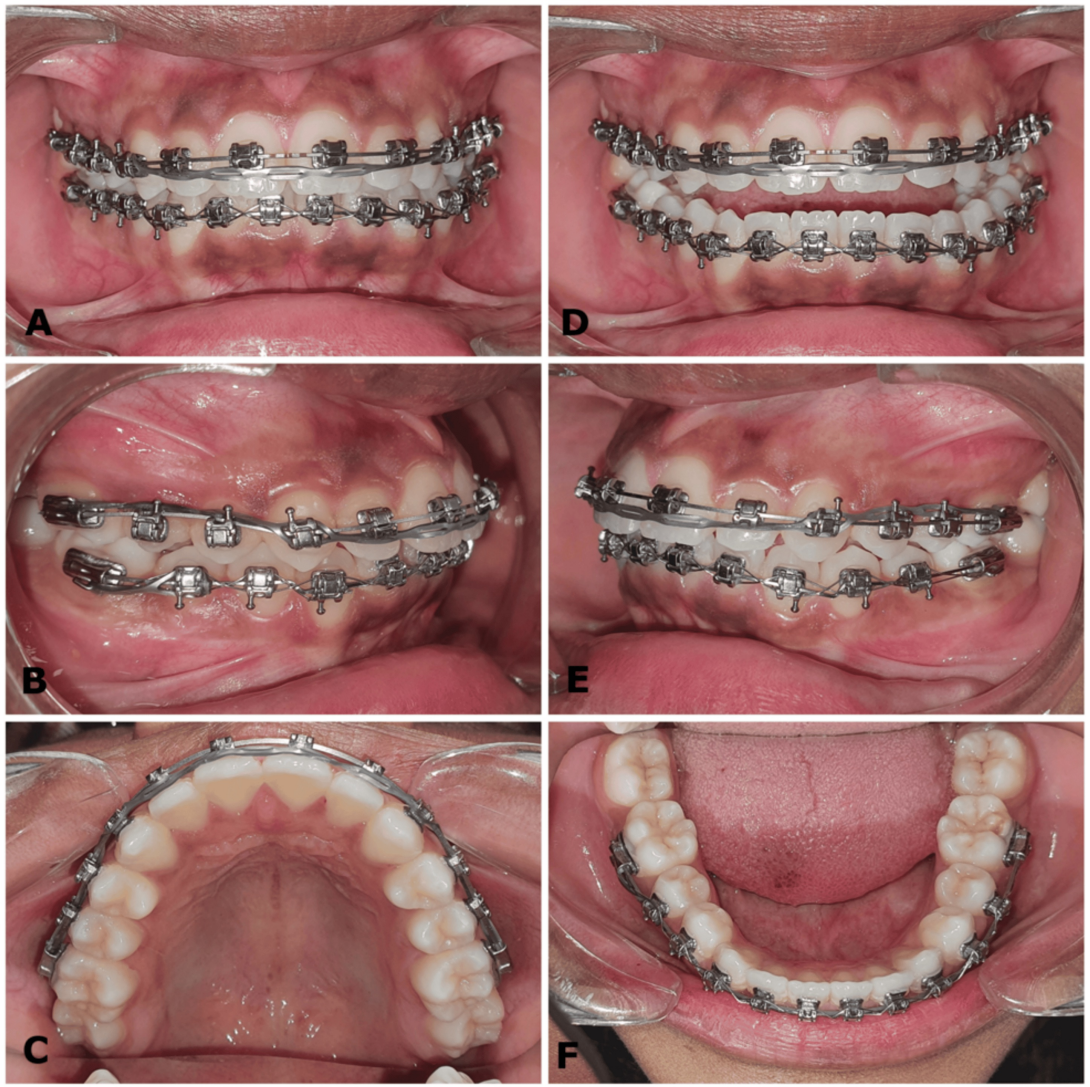
(A-F) Mid-treatment intraoral photographs with an elastomeric chain in the upper arch to prevent proclination and space opening of incisors

Bracket repositioning of upper incisor teeth was carried out by inversion to provide palatal crown torque. The upper archwire progressed from 0.016" x 0.022" superelastic NiTi to 0.017" x 0.025" superelastic NiTi. An elastic chain (Storino Leash) was applied in the upper arch from molar to molar to prevent and control incisor proclination (Figure 8).

**Figure 9:**
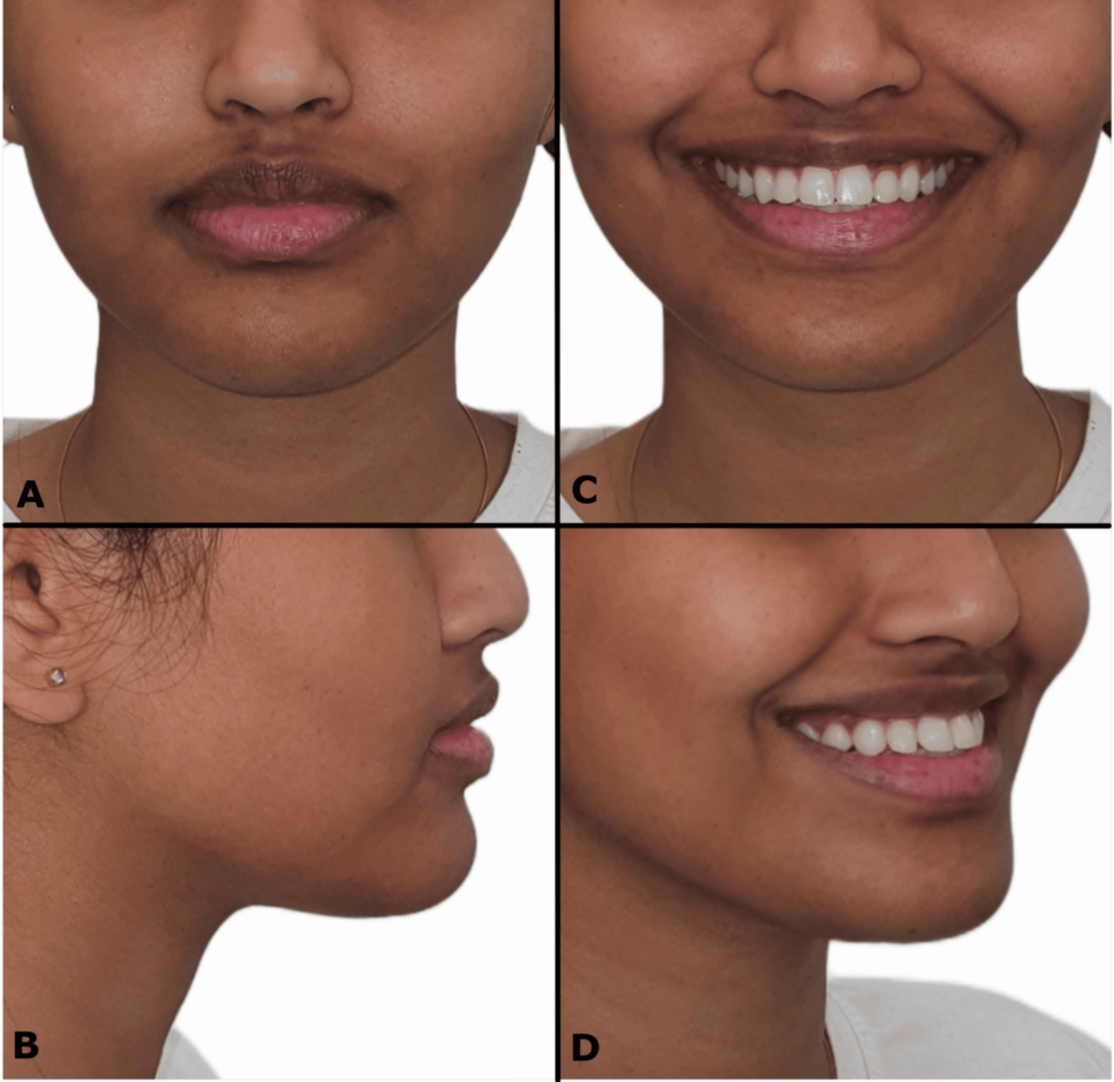
(A-D) Post-treatment extraoral photographs

**Figure 10:**
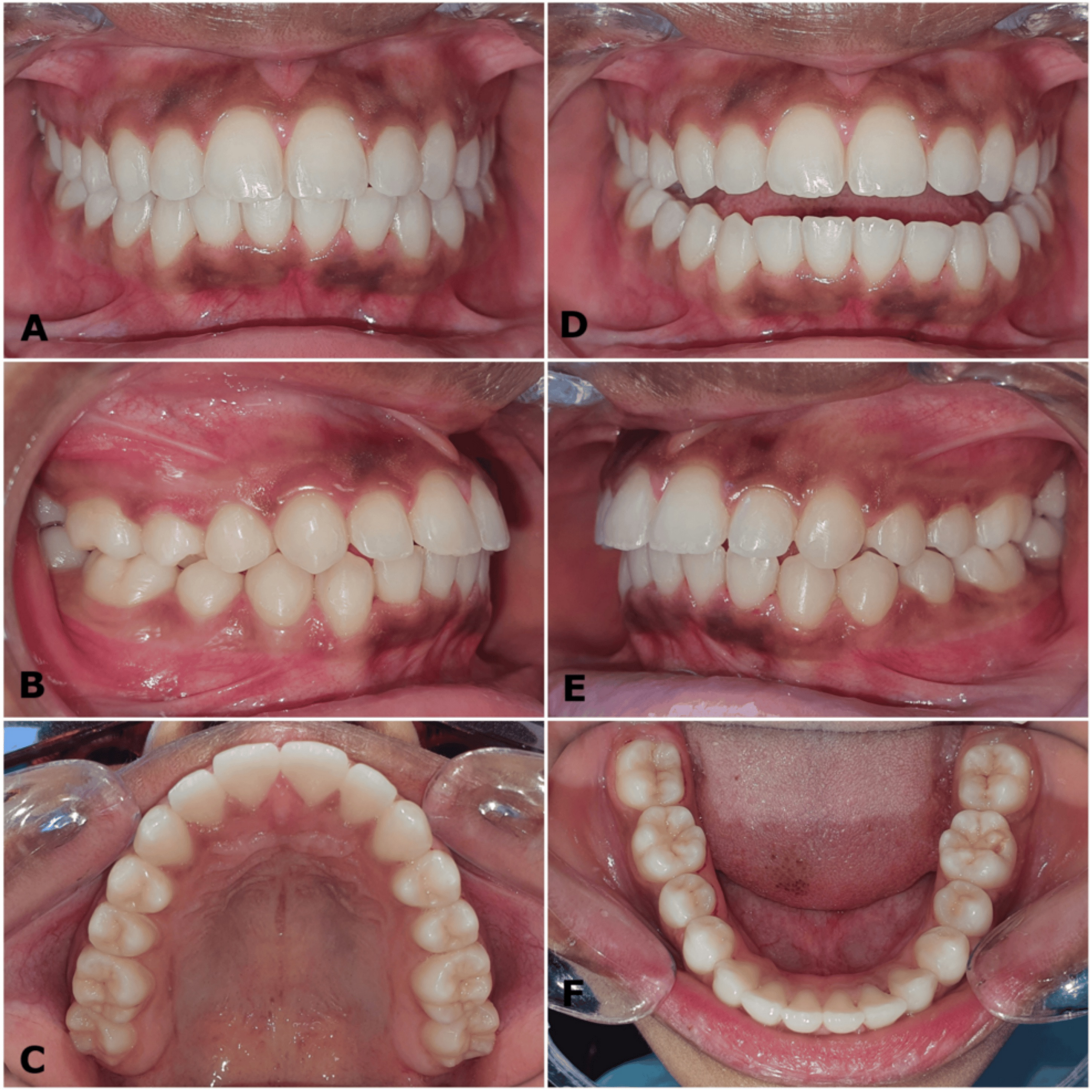
(A-F) Post-treatment intraoral photographs

**Figure 11:**
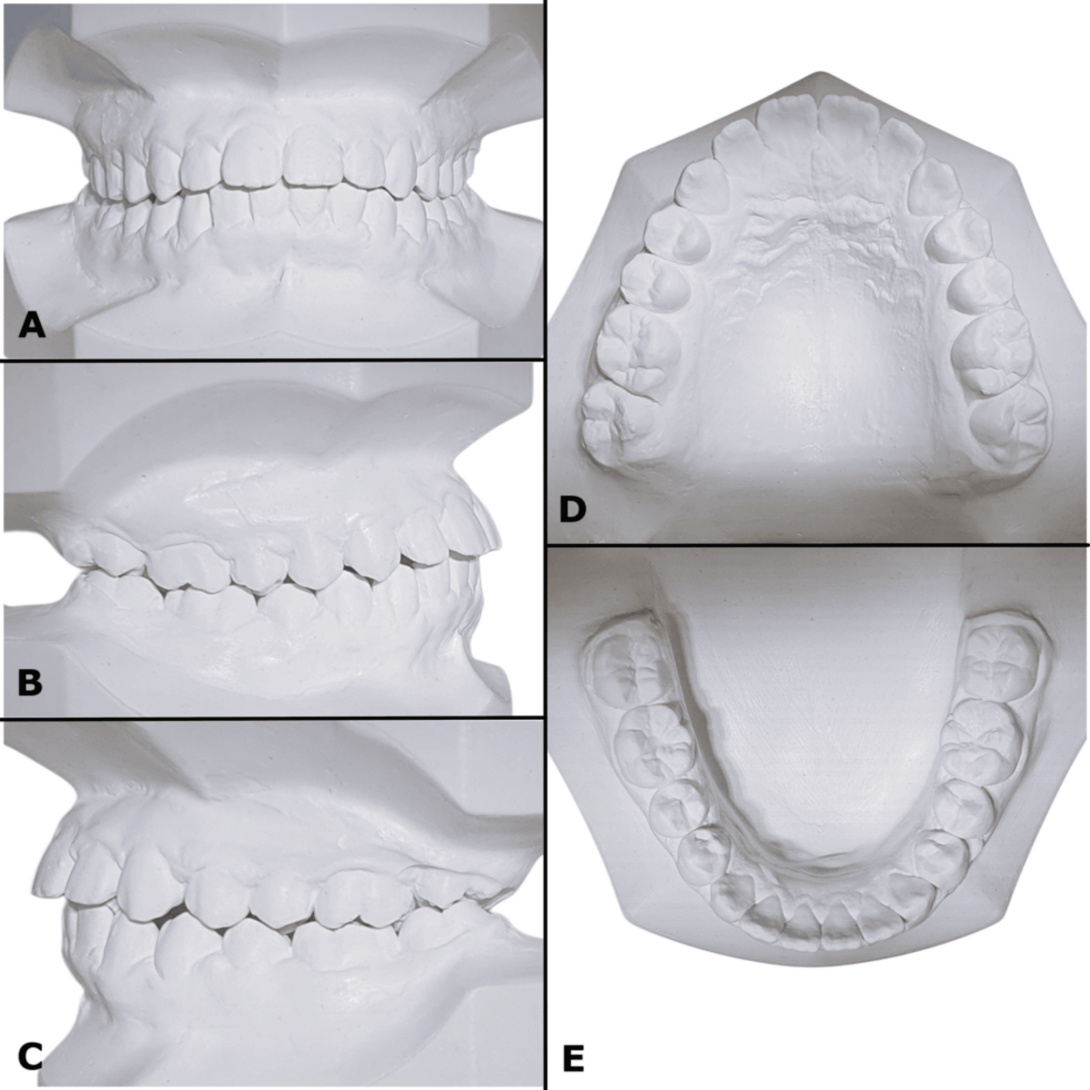
(A-E) Post-treatment study models

**Figure 12:**
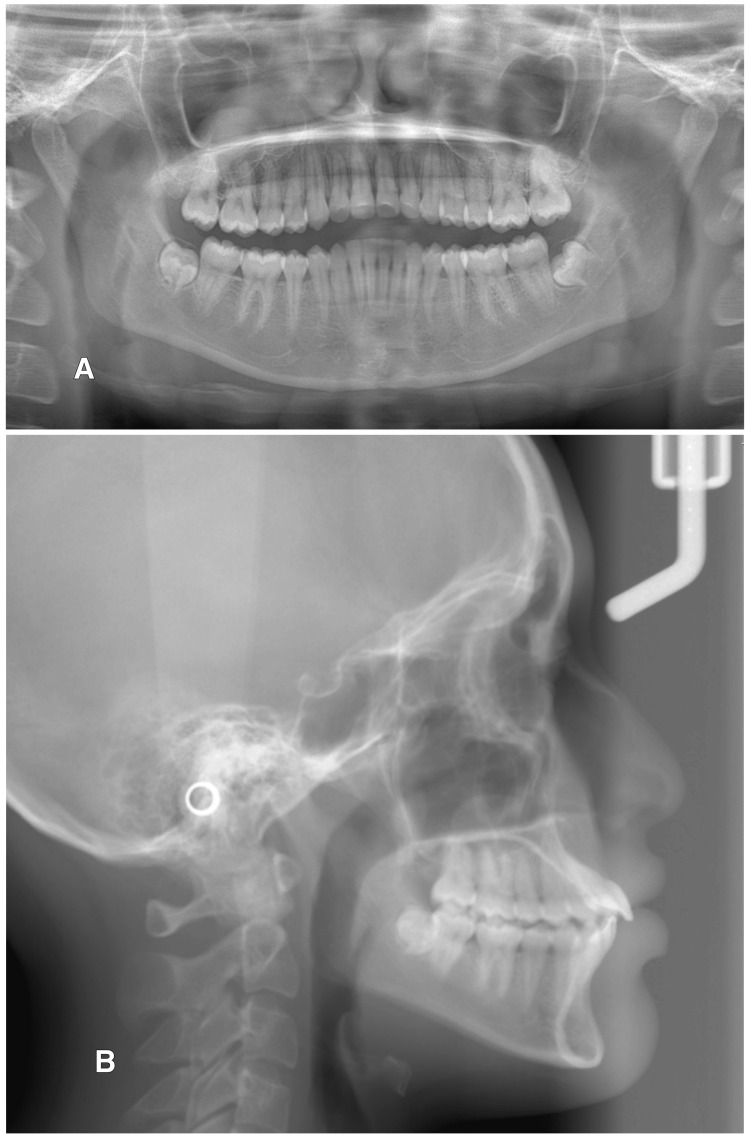
Post-treatment radiographs: (A) panoramic radiograph and (B) lateral cephalogram

**Figure 13:**
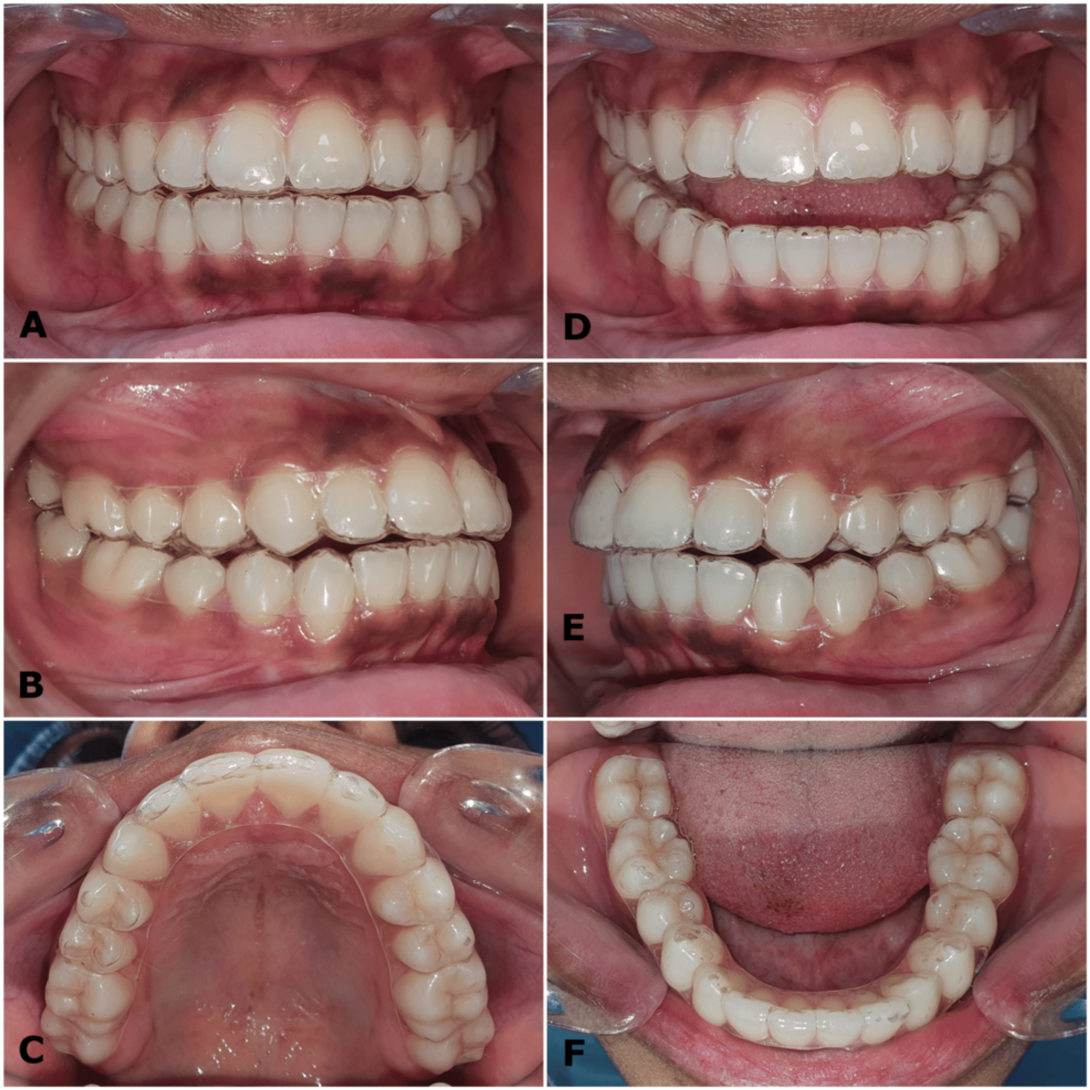
(A-F) Upper and lower arches with full-coverage vacuum-formed clear plastic retainers

In November 2023, the lower archwire progressed from 0.016" x 0.022" to 0.017" x 0.025" NiTi archwire with continued intrusion bends incorporated distal to the canines using V-bend pliers and an upper archwire with 0.019" x 0.025" NiTi. In mid-December 2023, the upper and lower orthodontic appliances were debonded. End-of-treatment extraoral photographs (Figure 9), intraoral photographs (Figure 10), study models (Figure 11), and radiographs (panoramic and lateral cephalometric radiographs) (Figure 12) were obtained three months following the completion of active orthodontic treatment. Retention was achieved with the application of full-coverage vacuum-formed retainers for both the maxillary and mandibular arches (Figure 13). The patient was delivered with an additional set of aligners.

Treatment results

In accordance with the patient's prior request to complete orthodontic treatment within three months owing to travel, debonding of the orthodontic device was performed. Satisfactory outcomes were attained within a constrained timeframe of three months. An Angle class I molar relationship was maintained, with optimal anterior overjet and overbite, and enhanced facial aesthetics were achieved. A class I canine relationship was maintained on the right side, while the left canine relationship showed relative improvement. The maxillary and mandibular dental midlines were maintained. Crowding in the lower arch was corrected, along with the crossbites and anterior deep bite. Crossbite and crowding were corrected through leveling and alignment. The anterior deep bite was corrected with intrusion bends in lower archwires. A notable enhancement in smile aesthetics was achieved within a limited timeframe. The reverse curve of the smile was corrected, resulting in a consonant smile arc (Figure 10).

**Figure 14:**
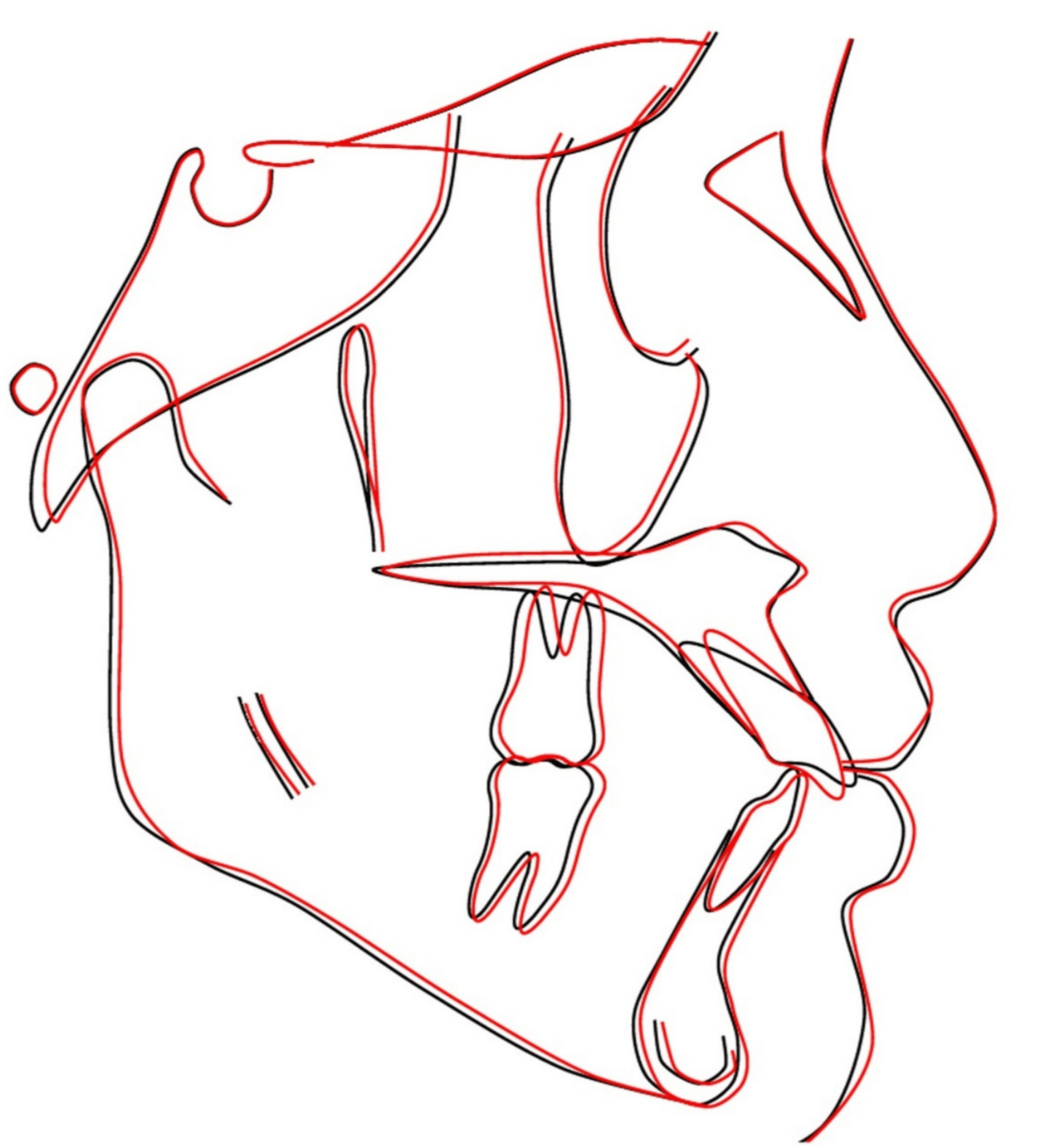
Pre-treatment and post-treatment cephalometric superimpositions (black tracing lines represent pre-treatment; red tracing lines represent post-treatment)

Post-treatment panoramic radiograph revealed improved root parallelism and no noted root resorption or pathology (Figure 12). Post-treatment cephalometric tracing analysis (Table 1) and superimpositions (Figure 14) revealed substantial improvements in the inclination of upper incisors to the upper palatal plane (U1-PP) from 136° to 126° with a palatine plane angle, with a retraction of 4 mm, contributing to a significant improvement in the patient’s facial profile. There is a 1.5 mm increase in lower incisor proclination.

**Table 1:** Pre-treatment and post-treatment cephalometric analysis measurements SNA: Sella-Nasion-A; SNB: Sella-Nasion-B; ANB: A-Nasion-B; SN: Sella-Nasion; PP: palatal plane; FMA: Frankfort-mandibular plane angle; IMPA: incisor mandibular plane angle; U1 to PP: upper incisors to the upper palatal plane

Measurement	Normal	Initial	Final
SNA (°)	81 ± 3	83	83.5
SNB (°)	78 ± 3	80	80.5
ANB (°)	3 ± 2	3	3
Wits appraisal (mm)	-1 ± 1	4	3
SN to PP (°)	8 ± 3	5	5
FMA (°)	25	27	26.5
U1 to PP (°)	109 ± 6	136	126
IMPA (°)	93 ± 6	96	97.5
Upper lip to E-line (mm)	-3.2 ± 2.0	-3	-4
Lower lip to E-line (mm)	3.0 ± 2.0	-3	-2

**Figure 15:**
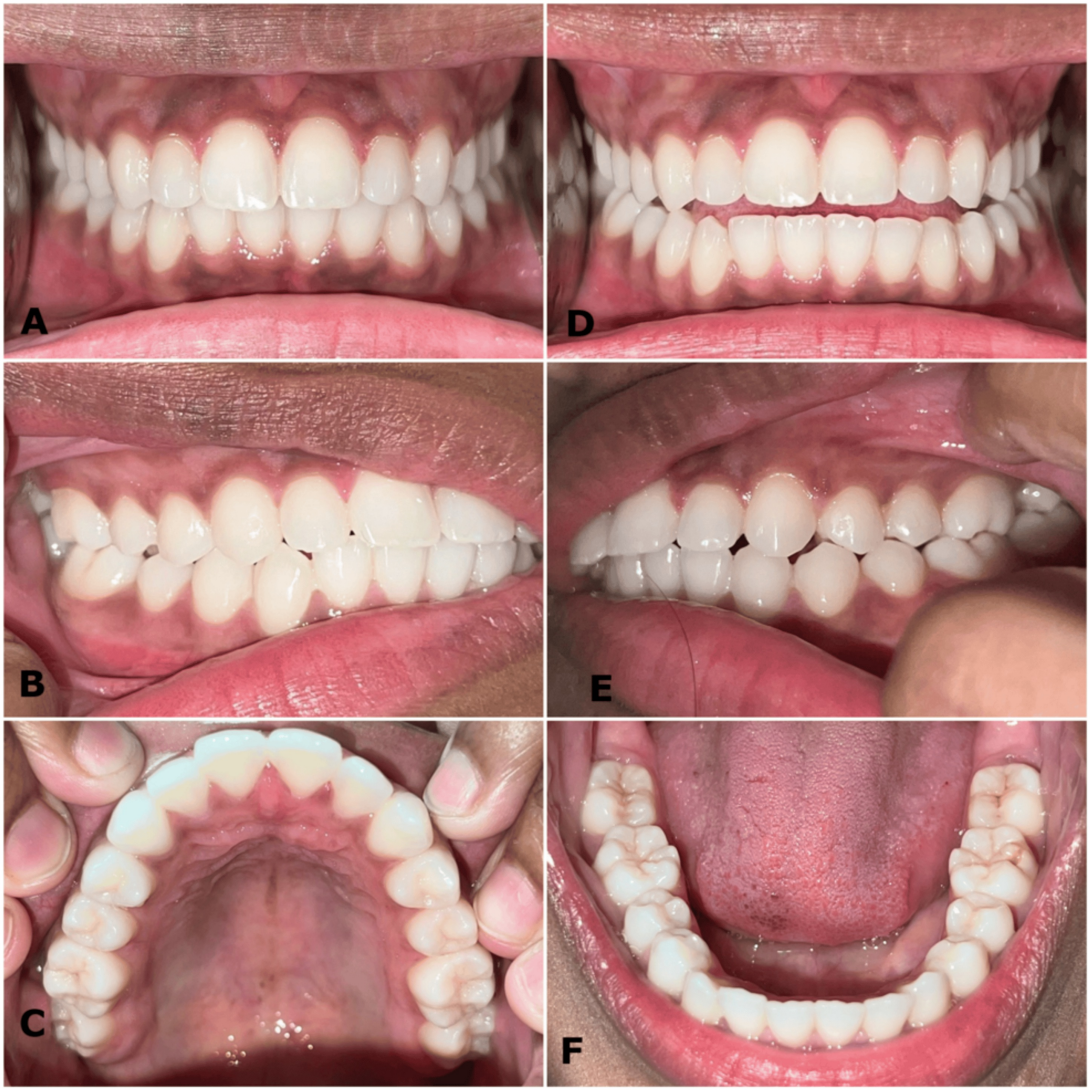
(A-F) One year and four months post-debonding intraoral photographs

At request, the patient consented and captured intraoral photos and sent them online, which were taken one year and four months into post-debonding; the photographs exhibit excellent maintenance of treatment results (Figure 15).

## Discussion

Orthodontic treatment, while highly effective in correcting dental malocclusions and improving oral health, often demands time and can span over several months to years. Extended treatment times can be exasperating for patients, resulting in lower compliance and increased dropout rates. Furthermore, lengthy treatments place a significant demand on orthodontic practices, affecting overall efficiency and patient turnover.

The appropriate appointment intervals can vary depending on several factors, including patient age, type of archwire and force-delivery system, periodontal health, extraction versus non-extraction cases, surgical or impacted tooth management, patient compliance, presence of decalcification or white spot lesions, risk of root resorption, and overall scheduling considerations [1]. Nevertheless, treatment times often exceed patient expectations. Surveys indicate that 40.8% of adolescent patients desired treatment to last less than six months, with 33.2% preferring between six and 12 months. Among adult patients, 42.9% preferred a treatment duration between six and 12 months, while 26.5% expected 12 to 18 months. Discrepancies between expected and actual treatment times contribute significantly to patient dissatisfaction [6]. Accurate estimation of orthodontic treatment time is essential, as it can improve cost transparency; mitigate the risk of undesirable effects such as root resorption, white spot lesions, dental caries, and gingival inflammation; and enhance both treatment success and patient satisfaction. Numerous techniques have been explored over the years to accelerate tooth movement, collectively termed accelerated orthodontics. These methods encompass pharmacological interventions like medications that expedite orthodontic tooth movement (OTM), including non-steroidal anti-inflammatory drugs (NSAIDs), acetaminophen, corticosteroids, bisphosphonates, herbal, and synthetic biomaterials; surgical procedures; physical orthodontic techniques; minimally invasive methods such as piezocision, discision, and micro-osteoperforation; and non-invasive techniques like cyclic vibrations, photobiomodulation, electric currents, and magnetic fields, which might reduce patient discomfort and reduce treatment duration and are aimed at achieving expedited outcomes.

Although the risk/benefit analysis suggests that extended appointment durations reduce overall chair time and overhead costs, enhance patient scheduling, minimize patients’ time away from school, and enable orthodontists to accommodate more patients, certain disadvantages may persist including white spots, carious lesions, gingival inflammation, patient compliance issues, and the potential for root resorption [7]. There may be missed opportunities to enhance and optimize tooth movement, potentially extending treatment duration. Unpredictable cancellations or missed appointments can disrupt the treatment plan and necessitate additional adjustments, resulting in delays [8]. Insufficient supervision of patients may lead to cases becoming intractable or overly corrected. The extended orthodontic treatment duration, which is usually around 20 to 30 months, has been found to adversely impact patient compliance, in addition to posing several risks such as root resorption, dental caries, enamel decalcifications, and periodontal problems [9,10]. It is recommended to perform periodic cleansing of brackets during archwire replacement with air polishing, effectively reducing debris levels and decreasing frictional forces [11]. In the realm of accelerating orthodontic treatment, it is essential to evaluate the wear protocols associated with clear aligner therapy. A study compared the effectiveness of three aligner wear protocols (seven-day, 10-day, and 14-day) on OTM. Although the 14-day protocol demonstrated slightly greater accuracy in certain posterior movements, the differences did not reach clinical significance. The seven-day protocol achieved similar clinical outcomes in half the treatment time, suggesting it is also a viable treatment option [12]. Another study compared 10-day and 14-day aligner change intervals and found similar OTM efficacy and pain perception between both groups. This depicts that aligner changes every 10 days can accelerate treatment without compromising clinical outcomes or patient comfort [13]. Expedited aligner wear protocols have been investigated at seven- or 10-day intervals for their potential to accelerate OTM compared to the conventional 14-day protocol. A systematic review of six studies, including randomized controlled trials (RCTs) and cohort studies, found no significant difference in OTM efficacy between expedited and conventional protocols, except for certain tooth movements, where the 14-day protocol showed superior outcomes. These findings suggest that a tailored, hybrid aligner wear protocol may be advantageous in complex cases [14].

The implementation of SLBs and low-friction (LF) ligating brackets has been suggested to reduce frictional resistance at the bracket-archwire-ligature interface, potentially enhancing OTM [15]. Nevertheless, research indicates that SLBs do not substantially reduce treatment duration due to binding and notching effects, which are present in both SLBs and conventional brackets [16]. Passive SLBs, which generate less friction than conventional brackets, may influence treatment duration [17]. The Damon system has effectively integrated LF brackets with light continuous forces (LCFs) from superelastic NiTi wires and aims to enhance comfort, decrease appointment frequency, minimize treatment duration, and enhance aesthetic results [18]. Retrospective studies indicate that passive SLBs may decrease treatment duration by as much as six months and reduce the number of treatment visits by four [19]. The influence of sliding mechanics and the regional acceleratory phenomenon (RAP) in canine retraction following extractions has received limited study regarding its effect on shortening treatment duration until a study proposed the Jiyugaoka Enjoyable Treatment (JET) system. It was a novel approach designed to shorten the orthodontic treatment duration using RAP, LCFs, and LF mechanics in extraction scenarios. In a 15-year-old patient with class I bimaxillary protrusion and crowding, the extraction of four premolars triggered the RAP and accelerated treatment. Passive SLBs and superelastic NiTi coil springs were employed to exert forces below 50 gf, with monthly adjustments. The total treatment duration was 13 months, showing minimal anchorage loss and minor root resorption, with excellent stability at the three-year and six-month follow-up. They aimed to accelerate tooth movements while socket remodeling was underway. Hence, the tooth is extracted, and the orthodontic appliances are bonded on consecutive days to initiate immediate tooth movements. The JET system may reduce the treatment time without considerable negative consequences [15].

Beyond a specific threshold of force, biological response reaches saturation, and higher forces result in no further increase in inflammatory markers, osteoclasts, nor the amount of tooth movement. Therefore, higher forces to accelerate the rate of tooth movement are not justified. Inflammatory markers play an important role during tooth movement by controlling the rate of osteoclast formation and, therefore, bone resorption. While it may be presumed that augmenting orthodontic forces may increase the expression of inflammatory markers and the rate of tooth movement, evidence indicates that, in response to a higher magnitude of forces, a saturation point in the biological response is reached, beyond which no further increase in inflammatory markers or tooth movement is observed. Therefore, higher forces to increase the rate of tooth movement are not justified, and alternative methods should be considered [20]. Identifying patients who will benefit from shorter appointment intervals is essential. Orthodontists significantly influence the duration of orthodontic treatment, particularly considering their education and expertise, treatment planning, standards of care, and the level of quality required for finishing. Contrary to what many techniques currently advocate, shorter intervals between appointments appear to contribute to facilitating controlled orthodontic treatment and result in shorter treatment durations [21]. Three goals for archwire sequencing are (1) to ensure patient comfort, (2) to optimize the potential of each archwire, and (3) to attain the final archwire as soon as possible [22].

The proposed new protocol for accelerated orthodontic treatment

The proposed new protocol for accelerated orthodontic treatment is as follows: (1) the interval between the appointments for orthodontic adjustment is every 10 days, (2) metal passive SLBs are utilized, and (3) a rapid archwire sequencing approach with gradual progression from light archwires to thicker ones is used. The archwire sequence should commence with 0.012" thermal or superelastic NiTi archwire. To initiate treatment, the lightest possible archwires should be utilized. Standard archwire progression should be followed with 0.012" thermal NiTi or superelastic NiTi, 0.014", 0.016", 0.018", 0.016" x 0.022" superelastic NiTi archwires and followed as necessary.

To implement the above protocol, the patient must have no comorbidities, not be on any medications and be clinically healthy, and be best committed to oral hygiene maintenance. It is recommended to perform periodic cleansing of brackets during archwire replacement with air polishing, effectively reducing debris levels and decreasing frictional forces [11]. After removing the archwire, the gates of SLBs must be opened to facilitate air polishing. This can be accomplished with or without the use of polishing powder (Video 1).

**Video 1:** Air polishing without polishing powder for cleansing orthodontic brackets

The efficacy of the new protocol depends on careful patient selection, accurate diagnosis, comprehensive treatment planning, and effective communication between the orthodontist and the patient. While this study concentrated on one patient, further research with larger sample sizes, longer follow-up durations, and standardized methods is essential to validate the protocol's effectiveness across different malocclusion classifications. Additional research into the specific mechanism of accelerated tooth movement employing the aforementioned technique is recommended. The newly implemented protocol for 10-day appointments commences with the utilization of the lightest archwires feasible to facilitate physiological tooth movement. It is recommended that even arches demonstrating optimal alignment should also begin with the lightest archwires for this purpose. At each subsequent visit, a progressive increase in archwire size to the next available option or retying the existing archwire is necessary. A progressive increase in archwire dimensions is critical to maintaining biologically acceptable force levels throughout orthodontic treatment. Abrupt transitions to substantially larger archwires can generate excessive forces, potentially leading to patient discomfort and adverse periodontal responses. In our clinical protocol, the transition from a 0.012" to a 0.014" diameter archwire represents a minimal incremental increase of 0.002" (approximately 0.05 mm) in diameter. This conservative progression facilitates the delivery of optimal orthodontic forces, promoting efficient and controlled tooth movement while minimizing the risk of iatrogenic effects. It is prudent to evaluate the patient at each consultation from the anterior perspective to observe any midline deviations, arch leveling, and improvement in smile aesthetics. This protocol can be incorporated with the JET system for situations involving extraction to expedite treatment outcomes [15].

## Conclusions

The proposed protocol for scheduling orthodontic adjustment appointments every 10 days employing metal passive SLBs combined with rapid archwire progression with a gradual increase in size facilitated expedited tooth movement, providing a safe and effective method to initiate accelerated treatment. This approach can be combined with other techniques like temporary anchorage devices, the JET system, and other non-invasive acceleration methods, rendering it versatile and adaptive. Its success requires careful patient selection, accurate diagnosis, and effective communication between the orthodontist and the patient. Despite its potential, further research with larger sample sizes and a broader range of cases is required to substantiate its effectiveness.

The novel protocol with 10-day appointment intervals offers significant advantages, including oversight of treatment progress, micro-supervision of oral hygiene, and regular review of the treatment plans and necessary adjustments as required. This facilitates the early identification and correction of deviations from treatment objectives, ensures the completion of orthodontic treatments, and fosters patient satisfaction. It collectively contributed to more effective management of treatment progress and patient compliance. It represents a safer option, free from the potential adverse effects associated with an invasive accelerated technique.
